# The prognostic and clinical significance of IFI44L aberrant downregulation in patients with oral squamous cell carcinoma

**DOI:** 10.1186/s12885-021-09058-y

**Published:** 2021-12-13

**Authors:** Deming Ou, Ying Wu

**Affiliations:** 1 Department of Stomatology, Panyu Central Hospital, Guangzhou, 511400 China; 2 Department of Stomatology, Foshan Hospital of Traditional Chinese Medicine, Foshan, 528000 China

**Keywords:** Oral squamous cell carcinoma, Differentially expressed genes (DEGs), RankComp, Relative expression orderings (REOs), Interferon induced protein 44 like (IFI44L)

## Abstract

**Background:**

It is a basic task in high-throughput gene expression profiling studies to identify differentially expressed genes (DEGs) between two phenotypes. RankComp, an algorithm, could analyze the highly stable within-sample relative expression orderings (REOs) of gene pairs in a particular type of human normal tissue that are widely reversed in the cancer condition, thereby detecting DEGs for individual disease samples measured by a particular platform.

**Methods:**

In the present study, Gene Expression Omnibus (GEO) Series (GSE) GSE75540, GSE138206 were downloaded from GEO, by analyzing DEGs in oral squamous cell carcinoma based on online datasets using the RankComp algorithm, using the Kaplan-Meier survival analysis and Cox regression analysis to survival analysis, Gene Set Enrichment Analysis (GSEA) to explore the potential molecular mechanisms underlying.

**Results:**

We identified 6 reverse gene pairs with stable REOs. All the 12 genes in these 6 reverse gene pairs have been reported to be associated with cancers. Notably, lower Interferon Induced Protein 44 Like (IFI44L) expression was associated with poorer overall survival (OS) and Disease-free survival (DFS) in oral squamous cell carcinoma patients, and IFI44L expression showed satisfactory predictive efficiency by receiver operating characteristic (ROC) curve. Moreover, low IFI44L expression was identified as risk factors for oral squamous cell carcinoma patients’ OS. IFI44L downregulation would lead to the activation of the FRS-mediated FGFR1, FGFR3, and downstream signaling pathways, and might play a role in the PI3K-FGFR cascades.

**Conclusions:**

Collectively, we identified 6 reverse gene pairs with stable REOs in oral squamous cell carcinoma, which might serve as gene signatures playing a role in the diagnosis in oral squamous cell carcinoma. Moreover, high expression of IFI44L, one of the DEGs in the 6 reverse gene pairs, might be associated with favorable prognosis in oral squamous cell carcinoma patients and serve as a tumor suppressor by acting on the FRS-mediated FGFR signaling.

**Supplementary Information:**

The online version contains supplementary material available at 10.1186/s12885-021-09058-y.

## Introduction

Head and neck cancer is the sixth most common malignant tumor in the world [1], and oral squamous cell carcinoma (OSCC) is the most common head and neck cancer [2]. There are more than 300,000 new cases of OSCC worldwide every year, and more than 140,000 patients die from OSCC every year [2, 3]. More importantly, the incidence of oral squamous cell carcinoma has been increasing in recent years [4–6]; however, for those who receive treatment with surgery and chemotherapy or radiation therapy, the five-year survival rate is still not ideal [7, 8]. Biomarkers could guide the selection of appropriate therapy by predicting disease activity and progression, by predicting which individuals will respond to a particular therapy, and by providing pharmacodynamic information to facilitate assessment of response to therapy [9, 10].

It is a basic task in high-throughput gene expression profiling studies to identify differentially expressed genes (DEGs) between two phenotypes. Nevertheless, it has proven difficult to identify DEGs that show slight differential expression between two phenotypes. In particular, it is hard to detect sufficient DEGs for future researches when the sample size is not large enough. However, it is often possible to find a variety of datasets related to the same biological questions from public repositories, including Gene Expression Omnibus (GEO) [11] and ArrayExpress [12]. By combining datasets generated by multiple laboratories, weak biological signals can be efficiently detected, thus improving statistical capacity. Nevertheless, direct combining of multiple datasets could be hindered by various random factors including measurement batch effects [13]. These problems also pose key obstacles for the analysis of transcriptional data in The Cancer Genome Atlas (TCGA) where there are many small-scale batches of data. Even if data is preprocessed, the measurement of highly sensitive samples cannot be applied to independent samples [13–16]. Considering these limitations, the clinical use of quantitative transcriptional characteristics is limited.

In order to make the best use of the information provided by different datasets, meta-analysis uses statistical methods to combine *p*-values [17], effect sizes [18, 19], ranks [20, 21] and other results from independent researches. However, due to small sample sizes and large heterogeneity, high false negative rates may occur [22]. The more complex hierarchical Bayesian method “borrows” the information of all genes to strengthen the inference of which genes are expressed differently [23–26]. Nevertheless, the crucial assumption of hierarchical models usually induces a bias to the estimation of gene differences [27]. Although batch effect adjustment methods have been used to normalize data across studies, the normalization process itself might lead to distortions of the true biological signals [28, 29] and even false inter-group differences, particularly when phenotypic groups are distributed unevenly across batches [14, 30]. To solve these problems, recently, Wang and colleagues have proposed RankComp, an algorithm, to analyze the highly stable within-sample relative expression orderings (REOs) of gene pairs in a particular type of human normal tissue that are widely reversed in the cancer condition, thereby detecting DEGs for individual disease samples measured by a particular platform [14, 31]. Since first reported, RankComp has been used to detect differentially expressed genes between different groups in lung, colorectal [31], and breast cancers [32] and osteosarcoma [33]. REOs of gene pairs are not sensitive to measurement batch effect [34] and quite consistent across distinct platforms [31], which facilitates RankComp to be used for cross-study comparison of gene expression.

**Fig. 1:**
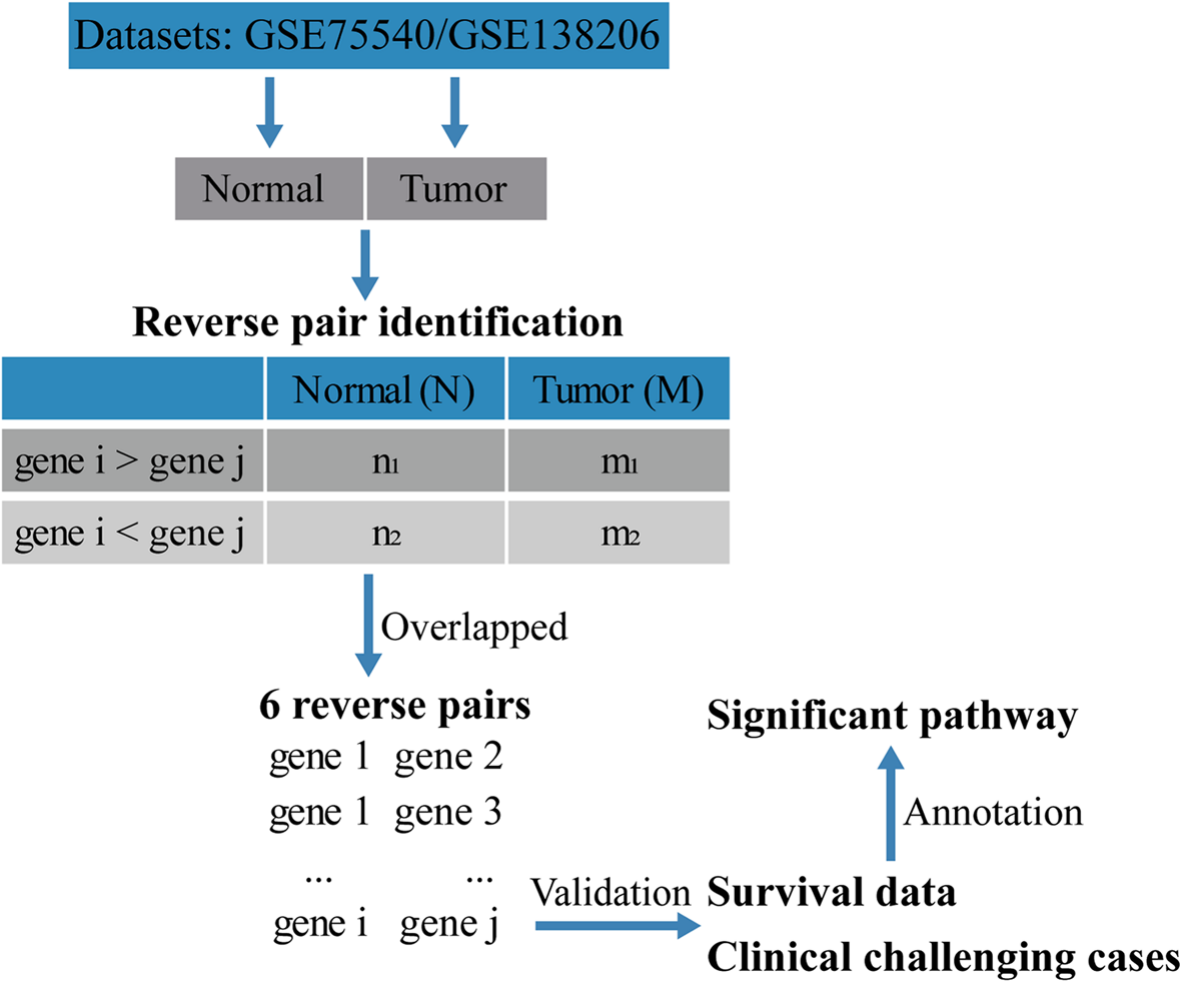
A schematic diagram showing the workflow of the present study. The workflow includes three major steps: the development of the REOs-based signature in the training datasets, the validation of the signature in validation datasets, and the signaling pathway enrichment annotation of the identified gene pairs

In the present study, by using RankComp algorithm based on training datasets GEO series GSE75540 and GSE138206, we attempted to identify differentially expressed gene pairs with highly stable REOs in oral squamous cell carcinoma and obtained 6 pairs of overlapping and stable reverse gene pairs. Through literature review, IFI44L, among the 6 gene pairs, was selected for further prognostic analysis and signaling pathway enrichment annotation (Fig. 1). Collectively, we confirmed the RankComp algorithm could identify reverse gene pairs with stable REOs in oral squamous cell carcinoma, providing potential prognostic markers and therapeutics targets for oral squamous cell carcinoma.

## Materials and methods

### Datasets and pre-processing

GSE75540, GSE138206 were downloaded from GEO (https://www.ncbi.nlm.nih.gov/geo/). GSE75540 contained the expression profile of oral tongue squamous cell carcinoma and adjacent normal tissues. GSE138206 contained expression profile of oral squamous cell carcinoma and adjacent normal tissues. GSE75540 was based on the Illumina HumanHT-12 V4.0 expression beadchip (gene symbol), Illumina HumanMethylation450 BeadChip [UBC enhanced annotation v1.0], and Illumina HumanHT-12 WG-DASL V4.0 R2 expression beadchip [gene symbol version] platforms. GSE138206 was based on the [HG-U133_Plus_2] Affymetrix Human Genome U133 Plus 2.0 Array platform.

The software we used in this study include Python (v3.7.6; https://www.python.org/downloads/release/python-376/) and R studio (v4.0.2; https://www.rstudio.com/).

### Stable REOs, the RankComp algorithm, and the concordance score

In each sample, the REO of a gene pair (A and B) is denoted as either GA > GB or GA < GB exclusively, where GA and GB represent the expression values of gene A and B, respectively. Stable gene pairs obtained from training sets can be used as markers of classification in any given sample. Based on these stable gene pairs, a classification model was constructed. For unclassified samples, the relative expression order relationships of all stable gene pairs were calculated. In a sample, the probability that GA < GB occurs less than or equal to k times in n pairs of stable gene pairs can be calculated by the following formula:

$$\boldsymbol{P}\left(\boldsymbol{k},\boldsymbol{n}\right)=\mathbf{1}-{\sum}_{\mathbf{i}-\mathbf{n}}^{\mathbf{k}-\mathbf{1}}\left(\begin{array}{c}\boldsymbol{n}\\ {}\boldsymbol{i}\end{array}\right)\mathbf{0}.{\mathbf{5}}^{\boldsymbol{i}}{\left(\mathbf{1}-\mathbf{0.5}\right)}^{\mathbf{n}-\mathbf{i}}$$

In the formula, n represents the number of samples and k represents the number of occurrences of GA < GB in n samples. If the *P* value is less than 0.05, it means that most of the samples can maintain the relationship of GA < GB, and we call it a significantly stable pair. For a sample with an unknown category, let k be the number of times that the genetic rank relationship of GA > GB appears in stable reversal pairs, and n is the logarithm of significantly stable pairs. Here, we define k as stable pair count. If P(k,n) < 0.05, then the sample is significantly in line with the characteristics of Type B and is classified as type B, and if P(nk,n) < 0.05, the sample is significantly in line with the characteristics of Type A and is classified as type A. In the present study, we use the reversal model of stable gene pairs for the classification of two types of samples. If GA < GB and GA > GB are significantly stable pairs in type A and type B, then we call (GA, GB) a stable reversal pair.

### Survival analysis

The correlation between IFI44L expression and the disease-free survival (DFS) in patients with oral squamous cell carcinoma was analyzed using the Kaplan-Meier survival analysis by grouping the cases in GSE4676 or GSE75540 taking the median expression values of IFI44L as cut-off. We used univariate and multivariate Cox regression analysis to identify clinical risk parameters associated with survival using GSE34115 (contained the gene expression profile of archival tongue squamous cell carcinoma), GSE42023 (contained the gene expression profile of archival tongue squamous cell carcinoma), GSE84846 (contained the gene expression profile of oral squamous cell carcinoma).

### Gene set enrichment analyses (GSEA)

To explore the potential molecular mechanisms underlying our constructed prognostic gene signature, GSEA (Gene Set Enrichment Analyses) [35, 36] was performed to find differential characteristics of oral squamous cell carcinoma patients with high or low IFI44L expression. *P* < 0.01 and FDR (false discovery rate) q < 0.05 were considered statistically significant.

## Results

### RankComp algorithm was used to construct reverse gene pairs in oral squamous cell carcinoma with stable REOs

On the basis of REO robustness, two datasets, GSE75540 and GSE138206, were used to analyze DEGs between healthy and cancerous samples and identify reverse gene pairs with stable REOs. As shown in Table 1, GSE75540 contained a total of 22,153 DEGs between 51 normal samples and 100 cancerous samples; top 500 up- or down-regulated DEGs could form 80 reverse gene pairs with stable REOs. GSE138206 contained a total of 7133 DEGs between 10 normal samples and 5 cancerous samples; top 500 up- or down-regulated DEGs could form 114 reverse gene pairs with stable REOs. These gene pairs intersected in 6 reverse gene pairs shown in Table 2.

**Table 1:** DEGs and reverse gene pairs with stable REOs

Dataset	Genes	Normal	Tumor	Reverse (top 500)	consist
GSE75540	22,153	51	100	80	6
GSE138206	7133	10	5	114

**Table 2:** Overlapping reverse gene pairs with stable REOs

Gene pairs	GeneA	GeneB
Pair 1	MSC	MMRN1
Pair 2	MMP9	TPM3
Pair 3	LAMB3	ALDH1A1
Pair 4	SCG5	ADH1B
Pair 5	IFI44L	NR4A2
Pair 6	HOXB2	ID4

### Functional annotations of overlapped reverse gene pairs

All the 12 DEGs in the 6 overlapped reverse gene pairs identified here have been reported to be associated with cancers. For example, Musculin (MSC) has been regarded as a component of a robust gene signature identified using a risk score model, and has been considered to be potential immunotherapy targets for hepatocellular carcinoma [37]. Multimerin-1 (MMRN1) has been recognized as a novel biomarker that may refine acute myelogenous leukemia risk stratification [38]. MMP-9 is known to be involved in carcinogenesis, inluding but not limited to invasive and metastatic abilities, and formation of blood vessels [39]. Tropomyosin 3 (TPM3) fusion with NTRK1 has been reported as one of the most well validated oncogenic events to date [40]. Laminin subunit beta-3 (LAMB3) relates to the invasion and metastasis of certain cancers, such as colon, pancreatic, pulmonary, cervical, gastric, and prostate cancer [41–43]. Aldehyde dehydrogenase 1 family member A1 (ALDH1A1) is a stemness marker and promotes the malignant behaviors in breast cancer [44, 45]. The low expression of secretogranin V (SCG5) could predict a poorer prognosis of pancreatic cancer [46]. Alcohol dehydrogenase 1B (ADH1B) polymorphisms have been reported to be associated with bladder cancer, gastric cancer, and breast cancer risk [47–49]. NR4A2 is a member of the Nur77 orphan receptor subfamily, which plays a critical role in human tumor cell survival [50–53]. HOXB2 serves as a tumor promotor in bladder cancer [54], colorectal cancer [55], and pancreatic cancer [56]. The oncogenic role of ID4 has also been reported in lung cancer [57], hepatocellular carcinoma [58], and breast cancer [59]. IFI44L serves as a tumor suppressor in hepatocellular carcinoma; in hepatocellular carcinoma patients, low IFI44L expression is associated with tumor size, recurrence, advanced stage and poor clinical survival [60].

### Prognostic potential of IFI44L

**Fig. 2:**
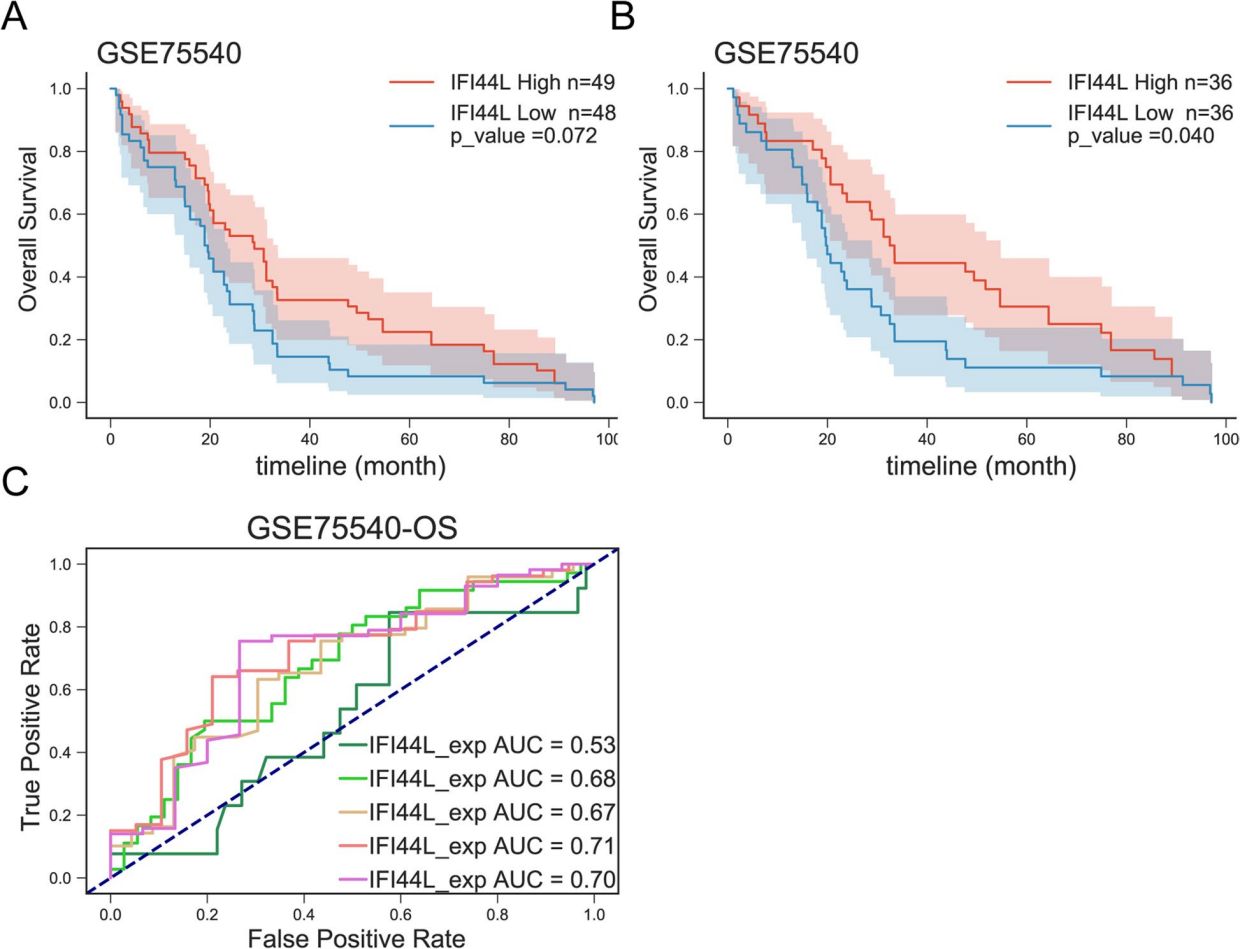
Correlation of IFI44L expression with the prognosis in patients with oral squamous cell carcinoma according to GSE75540 **A** Overall survival analysis on cancer tissues (*n* = 72) and peripheral blood samples (*n* = 25); 2 cancerous tissues with no survival information and 1 case with an overall survival of less than 30 days were excluded. **B** overall survival analysis on cancer tissues (n = 72). **C** ROC curves

To further investigate the clinical potential of the 12 DEGs, we analyzed the association of the 12 DEGs expression and the prognosis in oral squamous cell carcinoma patients. Kaplan-Meier survival analysis found that only IFI44L of the 12 DEGs was significantly associated with overall survival in patients with oral squamous cell carcinoma (*P* < 0.05; Table 3). GSE75540 included three types of samples: 75 cancerous tissues, 51 para-cancerous tissues, and 25 peripheral blood samples, for a total of 151 cases. In survival analysis, 51 para-cancerous tissues, 2 cancerous tissues with no survival information and 1 case with an overall survival of less than 30 days were first excluded. Thus, 72 cases of tissue samples and 25 cases of peripheral blood samples were assigned into high- or low-IFI44L expression group; however, although cases with higher IFI44L expression seemed to obtain better overall survival, the *p* value was > 0.05 (Fig. 2A). Considering that peripheral blood samples may differ from tissues and affect the analysis, peripheral blood samples were also excluded for the survival analysis. Finally, a total of 72 cases were included in survival analysis and assigned into low-IFI44L expression group and high-IFI44L expression group based on the median IFI44L expression; the Kaplan-Meier survival analysis showed that lower IFI44L expression was associated with poorer OS in oral squamous cell carcinoma patients (Fig. 2B). Then, we employed the receiver operating characteristic (ROC) curve [61] to test the prediction efficiency of the IFI44L. As shown in Fig. 2C-D, the area under the curve (AUC) for 3-,4-,5 years of OS were 0.69, 0.73 and 0.72, and for DFS were 0.70, 0.72, and 0.70. As revealed by the ROC curve, the IFI44L expression-based curve showed satisfactory predictive efficiency. In a larger cohort based on TCGA-HNSC data, lower IFI44L expression was associated with poorer OS (Fig. S1).

**Table 3:** IFI44L was significantly associated with overall survival in patients with oral squamous cell carcinoma

Gene	logRank.Pvalue
***IFI44L***	***0.040380449***
MSC	0.144207782
SCG5	0.240076311
HOXB2	0.301325148
LAMB3	0.364615339
TPM3	0.414847351
NR4A2	0.549182715
MMP9	0.639113275
ALDH1A1	0.718916123
ADH1B	0.825081505
MMRN1	0.903768924
ID4	0.914215189

**Fig. 3:**
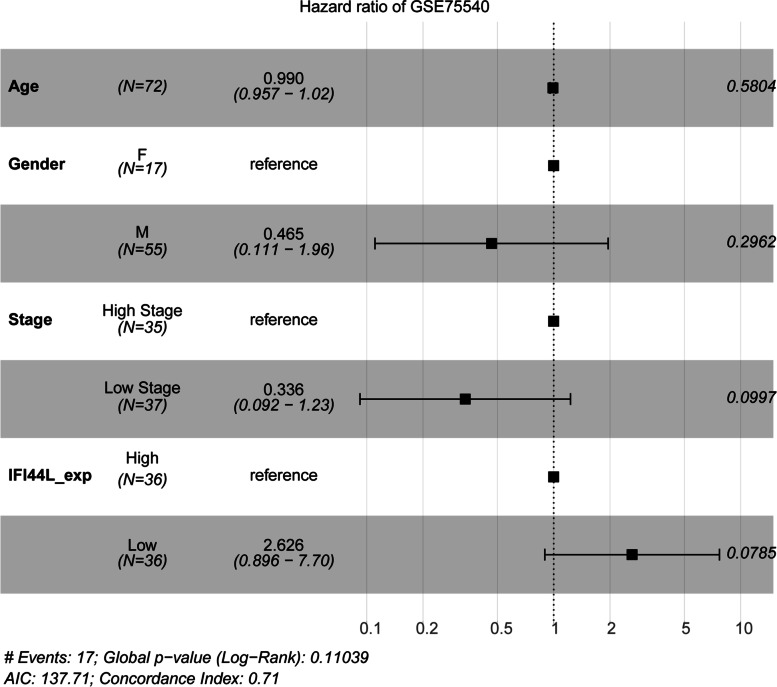
Univariate and multivariate Cox regression of oral squamous cell carcinoma patients

Moreover, based on the aforementioned 72 cases in GSE75540, we performed univariate and multivariate Cox regression analysis to analyze the association of age, gender, stage, and IFI44L expression with the OS in oral squamous cell carcinoma patients. As shown in Fig. 3 and Table 4, among these four factors, low IFI44L expression (HR = 2.63; 95% CI = 0.90-7.70) might predict higher risk for oral squamous cell carcinoma patients’ OS, although the *p* value was 0.0785. Based on TCGA-HNSC data, IFI44L is differentially expressed in subjects with different clinical parameters, including downregulated in male subjects (Fig. S2A), downregulated in subjects with tumor (Fig. S2B), downregulated in subjects with progression after therapy (Fig. S2C), and downregulated in higher tumor stages (not significantly, Fig. S2D).

**Table 4:** Univariate and multivariate Cox regression of oral squamous cell carcinoma patients

	Univariate		Multivariate	
	HR (95%CI)	p.value	HR (95%CI)	p.value
Age	1(0.96-1)	0.85	0.99(0.96-1.02)	0.5804
Gender	1.1(0.37-3.5)	0.82	0.47(0.11-1.96)	0.2962
Stage	0.42(0.15-1.1)	0.091	0.34(0.09-1.23)	0.0997
IFI44L_exp	2.9(1-8.1)	0.05	2.63(0.90-7.70)	0.0785

### Functional annotation of IFI44L

**Fig. 4:**
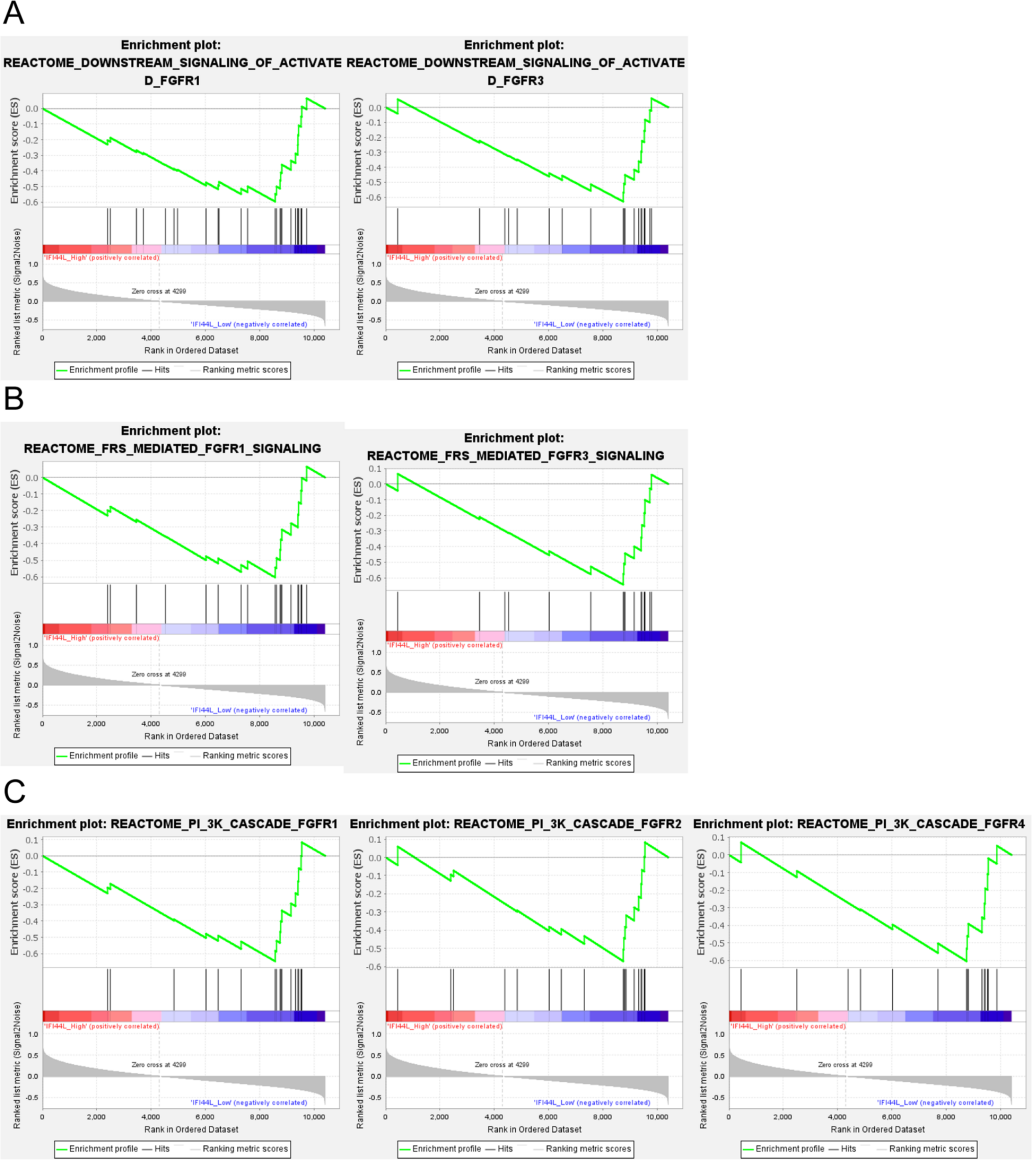
Gene Set Enrichment Analysis (GSEA) functional annotation analysis on IFI44L** A** The low expression of IFI44L activates FRS-mediated FGFR1 and FGFR3 signaling pathways. **B** The low expression of IFI44L activates the downstream signaling pathways of FGFR1 and FGFR3. **C** The low expression of IFI44L plays a role in the PI3K and FGFR cascades

Since low IFI44L expression showed to be associated with poor OS and DFS in oral squamous cell carcinoma patients, next, we performed GSEA functional annotation analysis on different characteristics in high- and low-IFI44L cases, attempting to identify signaling pathways related to IFI44L function. As shown in Fig. 4A-C, IFI44L downregulation would lead to the activation of the FRS-mediated FGFR1, FGFR3, and downstream signaling pathways; low IFI44L expression also plays a role in the PI3K-FGFR cascades.

## Discussion

In the present study, by analyzing DEGs in oral squamous cell carcinoma based on online datasets using the RankComp algorithm, we identified 6 reverse gene pairs with stable REOs. All the 12 genes in these 6 reverse gene pairs have been reported to be associated with cancers. Notably, lower IFI44L expression was associated with poorer OS and DFS in oral squamous cell carcinoma patients, and IFI44L expression showed satisfactory predictive efficiency by ROC curve. Moreover, low IFI44L expression were identified as risk factors for oral squamous cell carcinoma patients’ OS. IFI44L downregulation would lead to the activation of the FRS-mediated FGFR1, FGFR3, and downstream signaling pathways, and might play a role in the PI3K-FGFR cascades.

Totally different from traditional meta-analysis methods and batch-correction methods, RankComp, an algorithm based on the cross-platform significantly stable REOs for a particular normal tissue, is an economic and efficient method which can readily and accurately identify DEGs in any disease sample measured by any of the platforms [62]. Regarding other algorithms, both batch effect correction and normalization method might result in a distortion of true biological signals between two phenotypes, leading to false differences between groups [14, 28–30]; as for the RankComp algorithm, which has a high accuracy and is insensitive to measurement batch effect and data normalization, could normalize microarray samples measured by different platforms [62]. Herein, by using RankComp algorithm based on GSE75540 and GSE138206, we successfully identified 6 reverse gene pairs with stable REOs. As we have mentioned, all the 12 genes involved in the 6 reverse gene pairs have been reported to be associated with multiple cancers, suggesting that these reverse gene pairs might possess prognostic potential in oral squamous cell carcinoma.

**Fig. 5:**
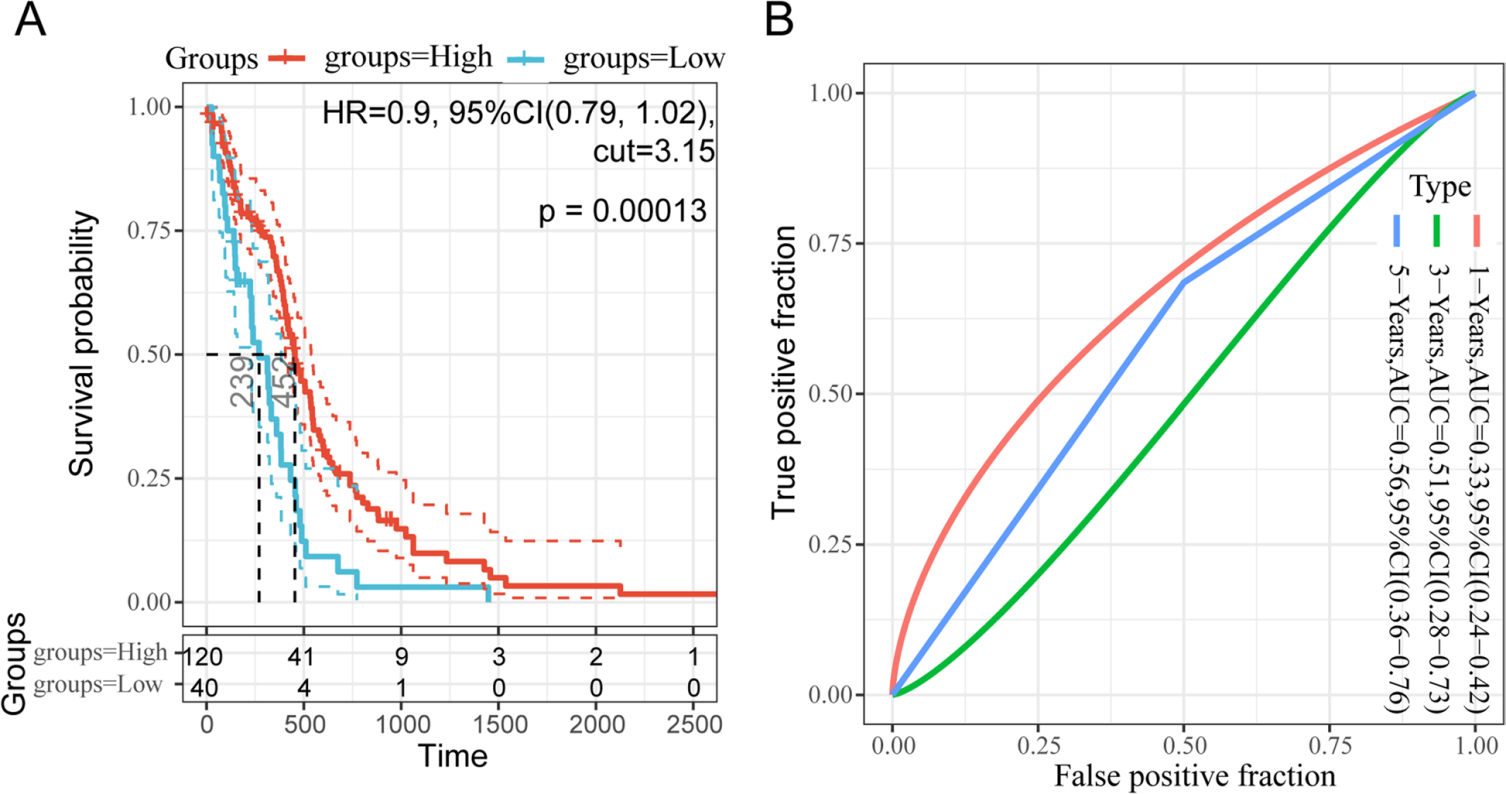
Sangerbox online analysis (http://sangerbox.com/) were performed to analyze the correlation of IFI44L expression with in glioma prognosis.** A** The correlation between IFI44L expression and survival probability of patients with oral squamous cell carcinoma. **B** The specificity and sensitivity of IFI44L expression being a prognostic marker

Among these 12 genes, little is known about IFI44L, which was found to exert moderate impact upon Hepatitis C virus infection [63]. Notably, the expression level of IFI44L has also been implicated in cancers [60, 64]. IFI44L has been recognized as a novel tumor-suppressor gene in human hepatocellular carcinoma that regulates met/Src signaling to affect cancer stemness, metastasis, and drug resistance [60]. However, the role of IFI44L in oral squamous cell carcinoma has never been investigated. Moreover, according to TCGA data, in glioma patients, higher IFI44L expression predicted higher survival probability (Fig. 5). Similarly, in the present study, according to GSE75540, lower IFI44L expression was associated with poorer OS and DFS in oral squamous cell carcinoma patients. Moreover, by using univariate and multivariate Cox regression analysis based on GSE75540, we identified the low IFI44L expression as a risk factor for oral squamous cell carcinoma patients’ OS. These data indicate that high IFI44L expression might be a favorable biomarker for oral squamous cell carcinoma patients.

Regarding possible molecular mechanism, GSEA analysis indicated that IFI44L downregulation would lead to the activation of the FRS-mediated FGFR1, FGFR3, and downstream signaling pathways; low IFI44L expression also plays a role in the PI3K-FGFR cascades. Increasing evidence demonstrated that FGFR aberrations are tied to oncogenesis, driving mutations where the acquisition of somatic molecular alterations could directly stimulate the growth and proliferation of tumor cells, promoting neovascularization and resistance to anticancer therapies [65–69]. The field of FGFR targeting has advanced rapidly with the recent development of new drugs repressing FGFs/FGFRs, thereby exhibiting a manageable safety profile in early clinical trials [70]. FGFR inhibitors have been reported to be effective in tumors with abnormal FGFR signaling, providing new treatment strategies within the era of precision medicine [71, 72]. Considering these previous findings, IFI44L might be a promising agent serving as a tumor suppressor in oral squamous cell carcinoma, possibly through acting on the FRS-mediated FGFR1, FGFR3, and downstream signaling pathways.

Collectively, we identified 6 reverse gene pairs with stable REOs in oral squamous cell carcinoma, which might serve as gene signatures playing a role in the diagnosis in oral squamous cell carcinoma. Moreover, high expression of IFI44L, one of the DEGs in the 6 reverse gene pairs, might be associated with favorable prognosis in oral squamous cell carcinoma patients and serve as a tumor suppressor by acting on the FRS-mediated FGFR signaling. Regarding the limitations of the present study, the RankComp algorithm might not be sufficient enough to identify genes whose differential expression results in slight alterations in the ranking. Moreover, based on the sensitivity of gene expression ordering to the microarray platforms to a certain extent, herein, the present study only analyzed the microarray data from the same platform. Future research should be performed to exclude gene pairs without stable ordering in datasets from multiple platforms, and identified promising factor should be investigated for specific effects in vitro and in vivo.

## Supplementary Information

**Additional file 1.**

**Additional file 2.**

## Data Availability

The datasets GSE75540, GSE138206 analysed during the current study are available in the GEO repository(https://www.ncbi.nlm.nih.gov/geo/).
