# Supplementary figures and images for: The prognostic and clinical significance of IFI44L aberrant downregulation in patients with oral squamous cell carcinoma
Source: BMC Cancer. 2021 Dec 13;21:1327. doi: 10.1186/s12885-021-09058-y (PMC8667451; doi:10.1186/s12885-021-09058-y)

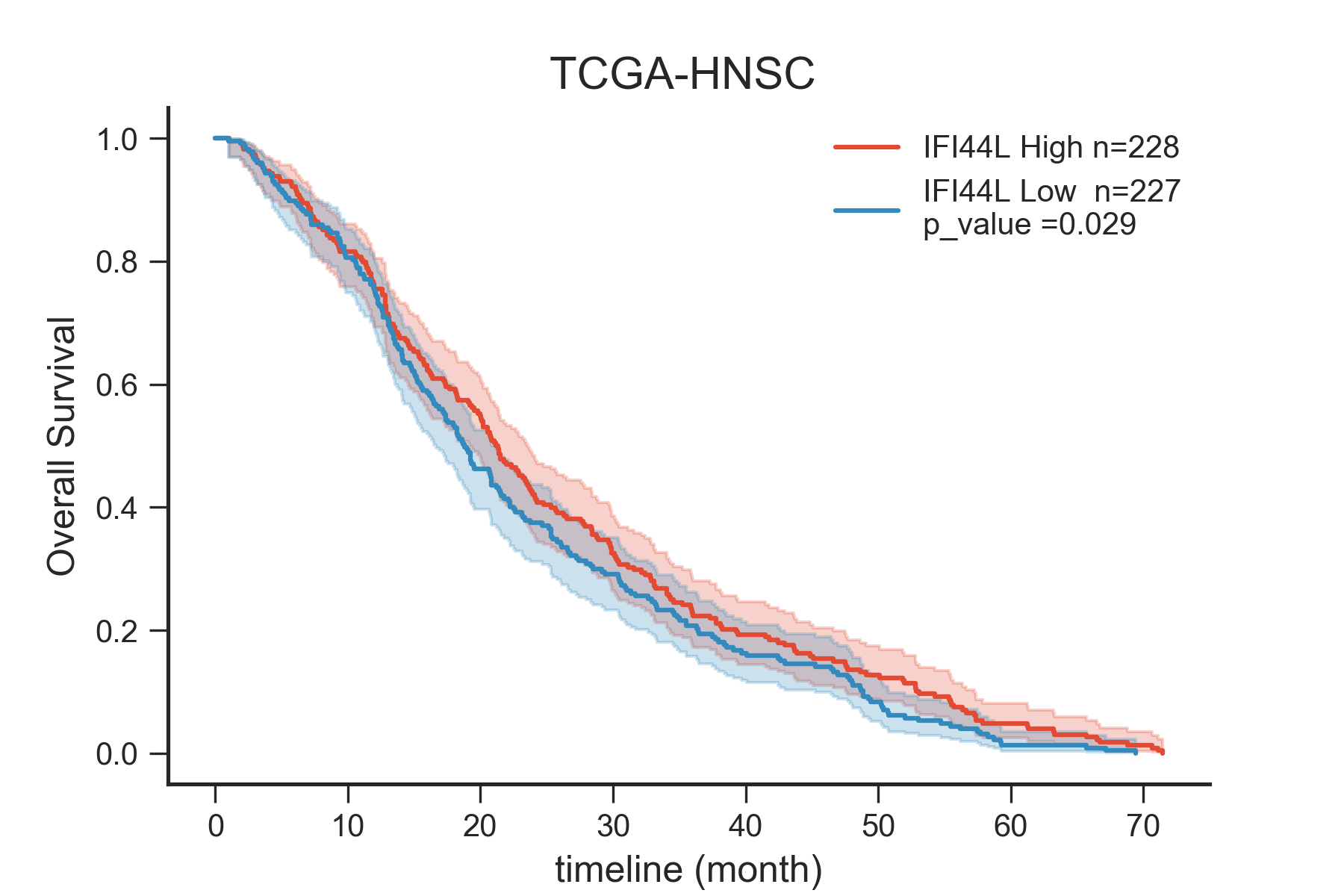

Supplement: Supplementary file 1 — Additional file 1. [file 12885_2021_9058_MOESM1_ESM.png]

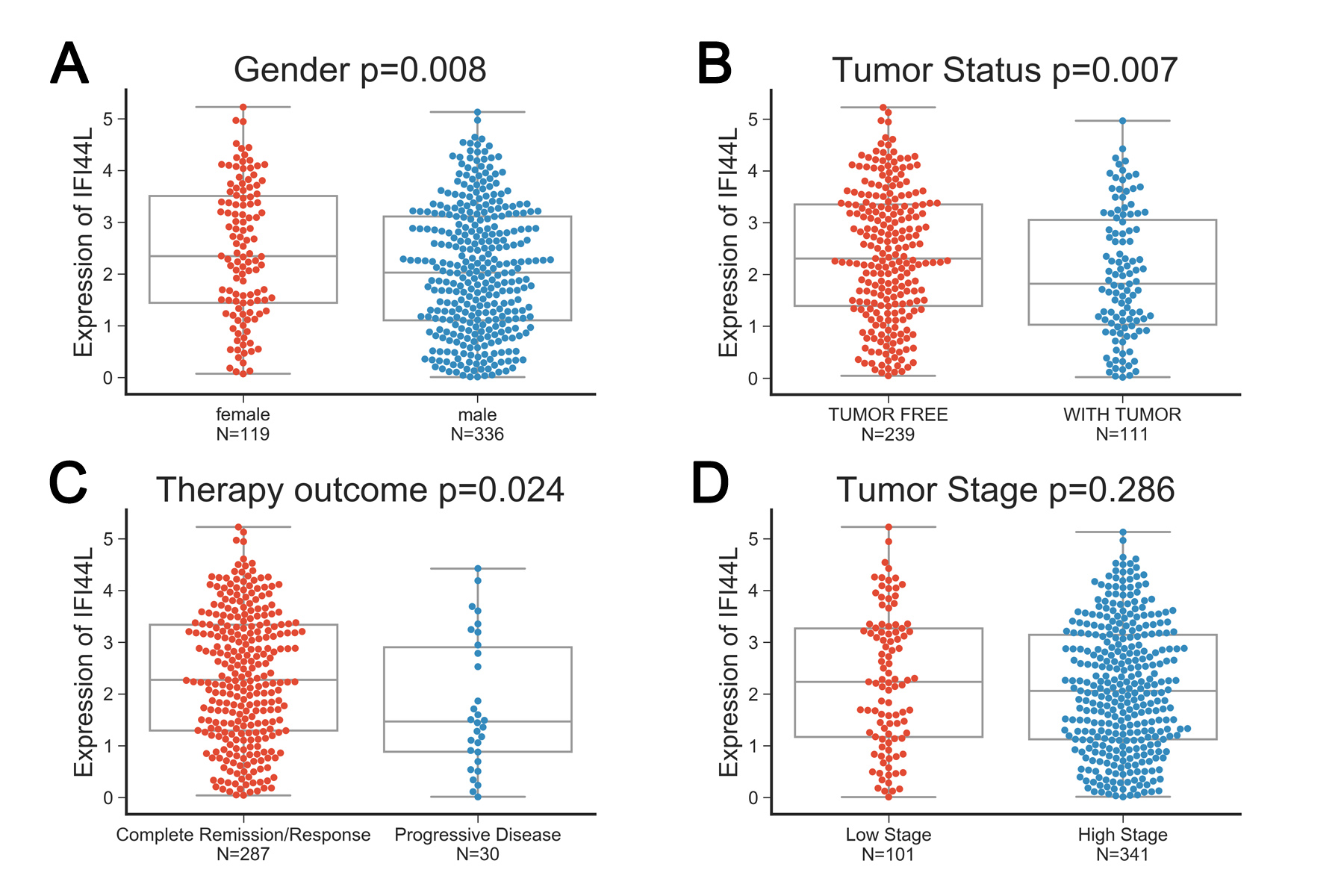

Supplement: Supplementary file 2 — Additional file 2. [file 12885_2021_9058_MOESM2_ESM.jpg]
